# A Longitudinal Study on Attenuated Structural Covariance in Patients With Somatic Symptom Disorder

**DOI:** 10.3389/fpsyt.2022.817527

**Published:** 2022-05-17

**Authors:** Hye Youn Park, Ye Eun Jang, Leonard Sunwoo, In-Young Yoon, Bumhee Park

**Affiliations:** ^1^Department of Psychiatry, Seoul National University Bundang Hospital, Seongnam, South Korea; ^2^Department of Psychiatry, Seoul National University College of Medicine, Seoul, South Korea; ^3^Human Rights Center, Hyupsung University, Hwaseong, South Korea; ^4^Department of Radiology, Seoul National University Bundang Hospital, Seongnam, South Korea; ^5^Department of Radiology, Seoul National University College of Medicine, Seoul, South Korea; ^6^Department of Biomedical Informatics, Ajou University School of Medicine, Suwon, South Korea; ^7^Office of Biostatistics, Medical Research Collaborating Center, Ajou Research Institute for Innovative Medicine, Ajou University Medical Center, Suwon, South Korea

**Keywords:** gray matter volume (GMV), magnetic resonance imaging, medically unexplained symptoms (MUS), structural covariance (SC), subtype

## Abstract

**Objective:**

This study was performed to investigate altered regional gray matter volume (rGMV) and structural covariance related to somatic symptom disorder (SSD) and longitudinal changes after treatment. Additionally, this study examined the relationships of structural alteration with its phenotypic subtypes.

**Methods:**

Forty-three unmedicated patients with SSD and thirty normal controls completed psychological questionnaires and neurocognitive tests, as well as brain magnetic resonance imaging. Voxel-based morphometry and structural covariances were compared between groups and between subgroups within the SSD group. After 6 months of treatment, SSD patients were followed up for assessments.

**Results:**

Patients with SSD exhibited attenuated structural covariances in the pallidal-cerebellar circuit (FDR < 0.05–0.1), as well as regions in the default mode and sensorimotor network (FDR < 0.2), compared to normal controls. The cerebellar rGMVs were negatively correlated with the severity of somatic symptoms. In subgroup analyses, patients with somatic pain showed denser structural covariances between the bilateral superior temporal pole and left angular gyrus, the left middle temporal pole and left angular gyrus, and the left amygdala and right inferior orbitofrontal gyrus, while patients with headache and dizziness had greater structural covariance between the right inferior temporal gyrus and right cerebellum (FDR < 0.1–0.2). After 6 months of treatment, patients showed improved symptoms, however there was no significant structural alteration.

**Conclusion:**

The findings suggest that attenuated structural covariance may link to dysfunctional brain network and vulnerability to SSD; they also suggested that specific brain regions and networks may contribute to different subtypes of SSD.

## Introduction

Somatic symptom disorder (SSD) as defined by the Diagnostic and Statistical Manual of Mental Disorders, Fifth Edition (DSM-5) (1), involves somatic disturbances associated with excessive thoughts and anxiety related to these somatic symptoms that disrupt the activities of daily life. While pain can be the predominant symptom in many patients with SSD, other patients may instead or also experience fatigue, palpitations, headache, nausea, indigestion, breathlessness, and dizziness (2), which have been classified as discrete symptom dimensions. Indeed, SSD shows diagnostic overlap within a broad range of other functional syndromes, including fibromyalgia, irritable bowel syndrome, functional neurological disorder, and somatoform dizziness, with diagnoses primarily based on consensus (3). These diagnoses are presumed to represent a common disease with different subtypes rather than multiple distinct diseases, where clinical phenotypes, diagnostic criteria, etiopathology, and treatment responses are similar among subtypes (3, 4). However, there is little concrete evidence to support this perspective. Moreover, some symptoms as chest pain, breathlessness and dizziness, have been understudied compared to somatic pain (2) and the neural circuits related to specific symptomatic presentations have rarely been investigated. Separate examinations of underlying brain networks for core psychological features of SSD and specific symptoms may produce additional evidence to address perspectives suggesting that SSD represents separate syndromes or subtypes of a single disease.

Previous studies have reported cognitive dysfunctions in psychomotor speed, attention, working memory, and executive functioning in patients with somatic symptoms (5). Other studies also found deficits in cognitive domains including processing speed, attention, memory, and executive functioning in patients with somatic symptom related disorders (6, 7). Recent studies have demonstrated that these cognitive dysfunctions are associated with depression as well as with somatic symptoms (7, 8). Indeed, indicators of emotional dysregulation such as depression, anxiety, anger, and alexithymia also frequently co-occur in patients with somatic symptoms (6, 9, 10), and these affective symptoms are known to affect cognitive functioning (11).

Regarding neurobiological aspects, previous neuroimaging studies on somatization disorder, somatoform disorder, and pain disorder, all of which fall under SSD in the DSM-5, have revealed altered neural circuitries involved in the development of SSD. Patients with SSD have consistently shown changes in the premotor and supplementary motor cortex, middle frontal gyrus (MFG), anterior cingulate cortex (ACC), insula and posterior cingulate cortex (PCC) in both structural and functional neuroimaging studies (12). In structural imaging studies of patients with SSD, morphological alterations and gray matter volume (GMV) changes have been observed in the caudate, ACC, MFG, angular gyrus, pituitary, amygdala, hypothalamus, fusiform gyrus, cuneus, inferior frontal gyrus, PCC and cerebellum (12–14). Previous studies have reported relationships of these brain structures, including the ACC, insula, prefrontal cortex, and amygdala, with psychopathologies that commonly occur in SSD such as alexithymia (15) and somatosensory amplification (2). Functional studies have also revealed disturbance of functional activity in the default mode network (DMN) (16, 17). Moreover, a recent study found aberrantly increased functional connectivity among the DMN, sensorimotor network (SMN), salience network, and dorsal attention network (DAN) in patients with SSD; these findings suggest that deficits in attention and perception are involved in SSD (18). However, few studies have investigated relationships between brain structural alterations, somatic symptoms, and cognitive functioning in patients with SSD. One study reported that a smaller mean GMV in the right MFG was correlated with more severe somatic symptoms and with impaired executive function in patients with somatization disorder (19). Another study reported executive dysfunction and cortical thinning of the prefrontal cortex in patients with chronic pain (20).

The relationships of changes in functional networks including DMN, SMN, salience network, and DAN or patterns caused by regional morphological alterations in patients with SSD remain unknown. Thus, the present study investigated patterns in brain anatomical structures in patients with SSD, by means of structural covariance analysis, which was recently introduced and has not yet been applied in this population (21). Structural covariance analysis can capture co-varied morphological characteristics in GMV or cortical thickness across brain areas, and these covariances partially overlap with functional connectivity because they are related to gray matter development and exhibit functional co-activation with neuroplasticity (21). Brain areas that co-vary in GMVs may be the part of relevant networks that subserve specific behavioral/cognitive symptoms and functions (22). Exploration of such structural covariance in SSD could provide complementary information regarding previous findings of functional magnetic resonance imaging (fMRI) studies, clarifying how inter-regional structures are reorganized with morphological changes and which functional connections and symptoms are interrelated. Symptomatic subtypes of SSD may also be newly differentiated through structural covariance analysis. Additionally, longitudinal investigation of structural brain changes has not been performed previously in patients with SSD.

We hypothesized that (1) unmedicated patients with SSD would show different structural covariance patterns compared to healthy controls, (2) structural covariance patterns would differ according to phenotypic subtypes of SSD, (3) structural alteration would be associated with cognitive functioning and clinical symptoms in patients with SSD, and (4) brain structural alterations would change after treatment. To address these hypotheses, we first compared structural covariances between patients with SSD and healthy controls. In comparing patient groups according to phenotypic differences, we also examined structural covaried regions in association with specific symptoms. Second, we evaluated the associations between individual regional gray matter volume (rGMV) and neurocognitive performance in SSD, which enabled exploration of the involvement of each cognitive function in relation to structural alteration of the brain in patients with SSD. Additionally, we performed follow-up assessments in patients with SSD after 6 months of treatment.

## Materials and Methods

### Subjects

Forty-three patients with SSD who presented to the Dizziness Center or the Pain Clinic of Seoul National University Bundang Hospital, Republic of Korea, and 30 normal controls were recruited from October 2017 to July 2019. The SSD diagnosis was confirmed by consensus between two clinical psychiatrists following clinical interviews based on the Structured Clinical Interview for DSM-5 Disorders-Clinician Version (23). The normal controls (healthy adults without psychiatric or medical illnesses) were matched for age and sex to the patients with SSD. The inclusion criteria were: (i) adults aged 20–64 years; (ii) confirmed diagnosis of SSD according to the DSM-5 criteria, including two or more somatic symptoms; and (iii) no use of psychotropic medication within the previous 2 months. The exclusion criteria were: (i) presence of new-onset neurological and/or medical disease with physical symptoms within the past 6 months; (ii) presence of any other mental disease, including major depressive disorder, bipolar disorder, a psychotic disorder, an anxiety disorder, a neurocognitive disorder, or a substance use disorder; (iii) history of brain injury and/or brain disease; and (iv) claustrophobia or presence of a non-MRI compatible device. As depressive and anxiety symptoms are common in patients with SSD, patients with such symptoms below the threshold for a clinical diagnosis were not excluded.

**FIGURE 1:**
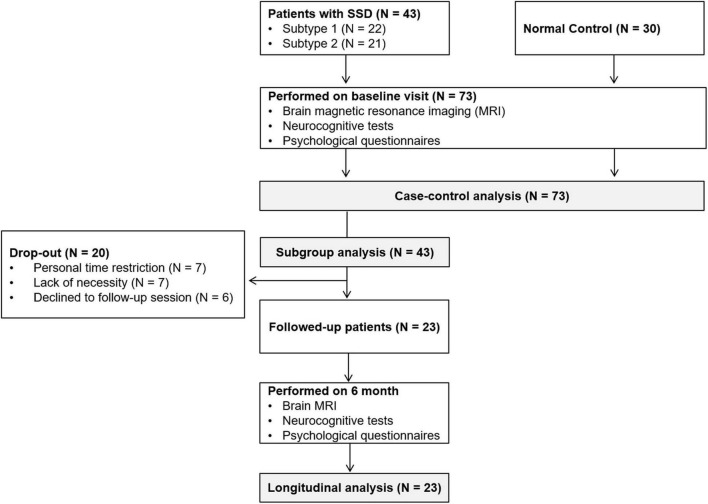
The flow chart of study protocol.

Sociodemographic data, psychological variables, neurocognitive functioning, and brain MRI data were collected from all participants in an initial assessment. For patients with SSD, treatment including pharmacological and non-pharmacological therapy was provided after the initial assessment. After 6 months, follow-up assessments of the patient group were performed to evaluate symptom and brain structural changes. Participants in the normal control group did not receive any specific intervention or undergo a follow-up assessment. The flow chart of study protocol is presented in Figure 1.

### Clinical and Neurocognitive Measures

After providing an informed consent, participants were asked to complete the following questionnaires: the Korean version of the Somatization Subscale in Symptom Checklist-90R (SCL-90-R-SOM) (24), Patient Health Questionnaire-15 (PHQ-15) (25), Beck Depression Inventory-II (BDI-II) (26), Beck Anxiety Inventory (BAI) (27), State–Trait Anger Expression Inventory (STAXI) (28), and Toronto Alexithymia Scale-20 (TAS-20) (29). For subgroup comparisons, we recruited patients in two symptom subgroups according to their main symptoms. Organ subtype classifications, previously suggested for inclusion as functional somatic syndromes and somatoform disorders were referenced (3). According to that classification, patients were sorted into two subtypes: subtype 1 (general symptoms; *n* = 22), and subtype 2 (musculoskeletal symptoms; *n* = 21). Additionally, neurocognitive functions were assessed using the Korean version of the Computerized Neurocognitive Function Test (CNT4.0, Maxmedica Inc., Seoul, Korea) (30) as follows: Auditory Verbal Learning Test (AVLT), Trail-Making Test (TMT), Wisconsin Card Sorting Test (WCST), Digit Span Test, Continuous Performance Test (CPT), and Verbal Fluency Test (VFT). We analyzed these measures as well as initial demographics (i.e., age and sex) using independent two sample *t*-tests (for continuous variables) and chi-square tests (for categorical variables). In the SSD group, clinical, neurocognitive, and brain structural variables were compared between pre- and post- treatment using the paired sample *t*-tests. Additionally, Pearson and Spearman correlation analyses were used to investigate the relationships between changes in the rGMV (delta; Δ = Post – Pre), neurocognitive functioning (Δ) and the clinical index (Δ). Statistical analyses were performed using the MATLAB software (MathWorks, Sherborn, MA, United States).

### Voxel-Based Morphometry Analysis

All MRI brain scans were obtained using a 3T Philips Achieva (Philips Healthcare, Inc., Best, Netherlands) with an 8-channel SENSE head coil at SNUBH. T1-weighted images were obtained using a fast field-echo three-dimensional imaging sequence with the following parameters: repetition time (TR), 8.1 ms; echo time (TE), 4.6 ms; flip angle (FA), 8°; field-of-view, 240 mm × 240 mm; acquisition matrix, 240 × 240; slice thickness, 1 mm; number of excitations, 1; scan time, 4 m 7 s.

To extract regional volumes from the entire brain, a Voxel-Based Morphometry (VBM) analysis was performed using the VBM-DARTEL procedure (SPM12; Wellcome Trust Centre for Neuroimaging, London, United Kingdom) (31), which provides clearer segmentation of tissues and better registration compared to a previously optimized VBM method (31).

No motion artifacts or other abnormalities were found on the T1-weighted images, which were inspected by a well-trained physician. The procedure for preprocessing T1-weighted images involved the following steps: (i) manual reorientation to the anterior commissure, (ii) gray matter segmentation based on a standard tissue probability map provided in SPM12, (iii) creation of a study-specific template, spatial normalization of individual images to the DARTEL template, and adjustment for volume signal changes during spatial normalization, and (iv) spatial smoothing of gray matter partitions using an 8 mm full-width at half-maximum Gaussian kernel. After preprocessing, rGMV was obtained by averaging the values in each area from 116 regions, which were defined using the Automated Anatomical Labeling Atlas (32).

### Structural Covariance Analysis

**FIGURE 2:**
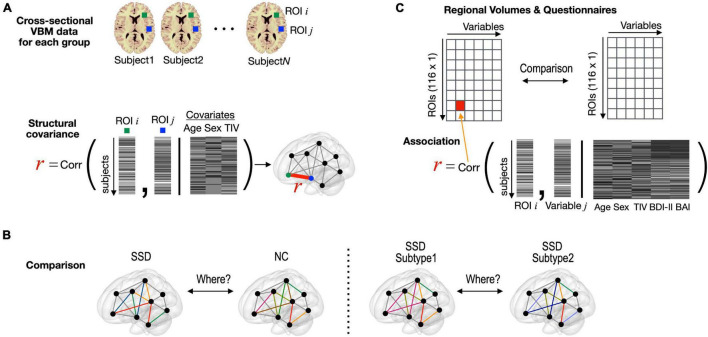
Statistical procedures used in this study. **(A)** Structural covariance between two regions was defined as partial correlation between two regional volumes (covariates: age, sex, and TIV), which were obtained from cross-sectional VBM data for each group. **(B)** Structural covariance was compared edge-by-edge between the entire SSD and normal control groups and between the subtype 1 and subtype 2 groups within SSD. **(C)** Partial correlation analysis with rGMV data and clinical variable. TIV, total intra-cranial volume; VBM, voxel-based morphometry; SSD, somatic symptom disorder; NC, normal control; ROI, region of interest; BDI-II, Beck Depression Inventory-II; BAI, Beck Anxiety Inventory.

In each group, we conducted partial correlation analyses between the rGMVs of two regions based on cross-sectional VBM data [covariates of age, sex, and total intra-cranial volume (TIV)] as a measure of structural covaried patterns (Figure 2A). Then, group-level inferences were applied using Fisher’s r-to-z transformation. For this, each correlation value was transformed to a normally distributed value, Z_*NC*_ or Z_*SSD*_ = 0.5 × [log(1 + R) – log(1-R)], and compared with Z = (Z_*NC*_ - Z_*SSD*_)/√[1/(*N*_*NC*_-3-*M*) + 1/(*N*_*SSD*_-3-*M*)], where *N*_*NC*_, *N*_*SSD*_, and *M* represent sample sizes for each group and the number of covariates used in the partial correlation analysis. Structural covariances were compared between the SSD group and normal controls (Figure 2B). The SSD group were further grouped as subtype 1 and subtype 2, and structural covariances and regional volume differences were compared between the two subtypes (Figure 2B). The rGMV data were correlated with the questionnaire data using partial correlation analysis, including the BDI-II and BAI scores as covariates, as well as age, sex, and TIV (Figure 2C).

All statistical analyses were performed using the MATLAB-based custom software (MathWorks, Sherborn, MA, United States). For inferences regarding structural covariance, several thresholds of the false discovery rate (FDR) = 0.05, 0.1, 0.15, and 0.2 were considered significant for addressing multiple-comparison issues (FDR < 0.05 indicated significance) (33). FDR thresholding controls the expected proportion of false positives only among brain areas exhibiting significant values (33). FDR control levels in the 0.1–0.2 range are considered practically acceptable, and several neuroimaging studies have applied them (34, 35).

## Results

### Demographic and Clinical Characteristics of the Participants

The demographic and clinical data are presented in Table 1. No significant differences in demographic characteristics were observed between the groups. Patients with SSD had significantly higher scores on questionnaires and exhibited poorer performance than the normal controls on the AVLT, CPT, and VFT (Table 1). Finally, all 22 patients in the subtype 1 group had dizziness with additional symptom as headache or concentration difficulties. All of 21 patients in the subtype 2 group had musculoskeletal pain including limb pain or backache or moving pain with additional symptom as pain on another place or tingling sensation. Three patients in the subtype 2 group had previously been diagnosed with fibromyalgia.

**TABLE 1:** Demographic and clinical characteristics, and results of neurocognitive tests.

	SSD (*N* = 43)	Normal Control (*N* = 30)		
	Mean (SD) or ratio	Mean (SD) or ratio	Statistics	*p*
**Demographic variables**				
Age, year	47.27 (11.43)	45.87 (9.22)	*t* = 0.56	0.57
Sex, Male/Female	13/30	9/21	χ^2^ = 0.0005	0.98
**Clinical variables**				
SCL-90-R-SOM	13.65 (7.83)	3.10 (3.25)	*t* = 6.96	< 0.001***
PHQ-15	13.07 (4.95)	3.03 (2.07)	*t* = 10.45	< 0.001***
BDI-II	15.76 (8.33)	3.66 (3.82)	*t* = 7.41	< 0.001***
BAI	20.76 (13.43)	2.87 (2.95)	*t* = 7.16	< 0.001***
STAXI-state	12.69 (5.08)	10.20 (0.48)	*t* = 2.68	0.009**
STAXI-trait	18.79 (5.15)	15.30 (4.27)	*t* = 3.05	0.003**
TAS-20	53.39 (10.81)	39.76 (8.64)	*t* = 5.74	< 0.001***
TIV (liters)	1.38 (0.10)	1.37 (0.11)	*t* = 0.34	0.73
**Neurocognitive tests**				
Auditory Verbal Learning Test	54.04 (7.72)	58.23 (5.88)	*t* = -2.50	0.015*
Trail Making Test -A response time (s)	25.55 (9.82)	22.80 (7.53)	*t* = 1.29	0.20
Trail Making Test-B response time (s)	44.83 (18.70)	42.10 (15.58)	*t* = 0.65	0.51
Wisconsin Card Sorting Test	4.83 (1.75)	4.96 (1.65)	*t* = -0.32	0.75
Digit Span Test- forward	7.49 (1.01)	7.65 (0.84)	*t* = -0.71	0.47
Digit Span Test- backward	5.79 (1.49)	6.13 (1.07)	*t* = -1.07	0.28
Visual CPT- correct	134.30 (1.75)	135.00 (0.00)	*t* = -2.18	0.033*
Visual CPT- overt	1.41 (2.42)	0.63 (0.71)	*t* = 1.72	0.090
Visual CPT- response time (s)	0.39 (0.05)	0.37 (0.02)	*t* = 1.55	0.12
Verbal Fluency Test	34.02 (10.59)	35.93 (10.43)	*t* = -0.76	0.44
Verbal Fluency Test- repeat	1.27 (1.50)	0.63 (0.92)	*t* = 2.09	0.040*

SSD, somatic symptom disorder; SD, standard deviation; SCL-90-R-SOM, Somatization subscale from Symptom Checklist-90-Revised; PHQ-15, Patient Health Questionnaire-15; BDI-II, Beck Depression Inventory-II; BAI, Beck Anxiety Inventory; STAXI-state, State-Trait Anger Expression Inventory-state; STAXI-trait, State-Trait Anger Expression Inventory-trait; TAS-20, Toronto Alexithymia Scale-20; TIV, total intra-cranial volume; CPT, Continuous Performance Test. All p-values are calculated with independent t-test (degree of freedom = 71 for all, 70 for Trail Making Test-A) or χ^2^ test. *p < 0.05, **p < 0.01, ***p < 0.001.

### Comparison of Structural Covariance Between Somatic Symptom Disorder and Normal Control Group

**FIGURE 3:**
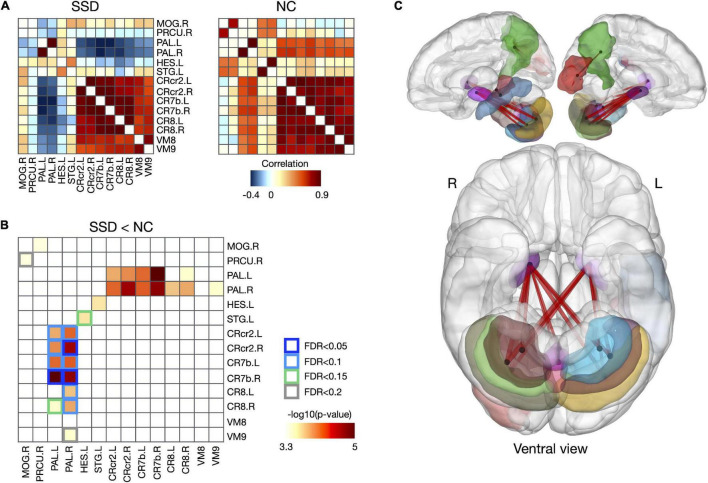
Disrupted structural covariance connectivity in patients with SSD. **(A)** Group-averaging structural covariance connectivity for SSD and NC group. **(B)** Reduced structural covariances and relating regions in patients with SSD, and **(C)** their 3D rendering. SSD, somatic symptom disorder; NC, normal control; MOG, middle occipital gyrus; PRCU, precuneus; PAL, pallidum; HES, Heschl’s gyrus; STG, superior temporal gyrus; CRcr2, cerebellar crus 2; CR7b, cerebellar lobe 7b; CR8, cerebellar lobe 8; VM8, vermis 8; VM 9, vermis 9; L, left; R, right.

Although the SSD group did not exhibit any significant changes in regional volume, it exhibited disrupted structures in multiple brain regions (i.e., changed structural covariances), compared with the normal control group. In the current study, structural covariances, which were centered at the bilateral pallidum and related to the cerebellum, were significantly decreased in the SSD group, compared with the normal control group (Figure 3 and Supplementary Table 1; FDR < 0.05–0.2). In detail, the regional volume of the bilateral pallidum showed altered connections with the cerebellar lobes including the bilateral crus 2, 7b, and 8 lobes, as well as the vermis 8 and 9 regions (Figures 3B,C and Supplementary Table 1). Reduced covariance was also found between the left Heschl’s gyrus and left superior temporal gyrus (STG), the right superior temporal pole (TPsup) and left dorsolateral superior frontal gyrus (SFGdor), and the right precuneus and right middle occipital gyrus (MOG) in patients with SSD.

### Correlations Between Regional Gray Matter Volumes and Clinical Variables

**FIGURE 4:**
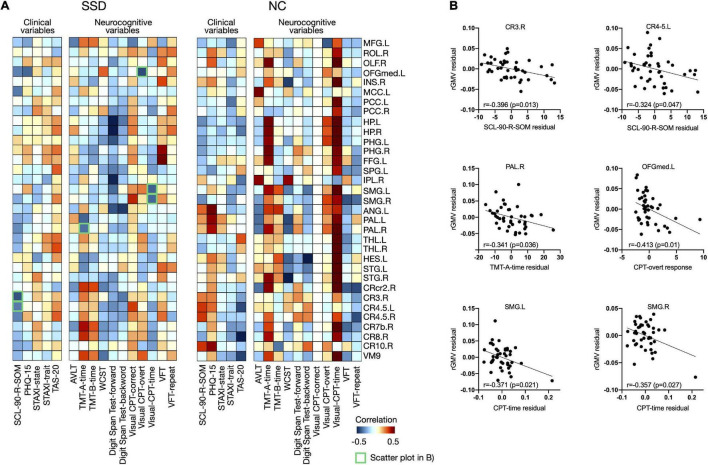
**(A)** Associations between clinical/neurocognitive variables and rGMVs in the whole brain for each group and **(B)** remarkable negative correlations among them, presented as scatter plots, are marked as green boxes in panel **(A)**. rGMV, regional gray matter volume; SSD, somatic symptom disorder; NC, normal control; MFG, middle frontal gyrus; ROL, Rolandic gyrus; OLF, olfactory cortex; OFGmed, medial orbitofrontal gyrus; INS, insula; MCC, middle cingulate cortex; PCC, posterior cingulate cortex; HP, hippocampus; PHG, parahippocampal gyrus; FFG, fusiform gyrus; SPG, superior parietal gyrus; IPL, inferior parietal lobule; SMG, supramarginal gyrus; ANG, angular gyrus; PAL, pallidum; THL, thalamus; HES, Heschl’s gyrus; STG, superior temporal gyrus; CRcr2, cerebellar crus 2; CR3, cerebellar lobe 3; CR4.5, cerebellar lobes 4–5; CR7b, cerebellar lobe 7b; CR8, cerebellar lobe 8; CR10, cerebellar lobe 10; VM9, vermis 9; L, left; R, right; SCL-90-R-SOM, Somatization subscale from Symptom Checklist-90-Revised; PHQ-15, Patient Health Questionnaire-15; STAXI, State-Trait Anger Expression Inventory; TAS-20, Toronto Alexithymia Scale-20; AVLT, Auditory Verbal Learning Test; TMT, Trail-Making Test; WCST, Wisconsin Card Sorting Test; CPT, Continuous Performance Test; VFT, Verbal Fluency Test.

Figure 4 shows the associations of psychological/neurocognitive variables with the rGMVs of multiple brain regions after controlling for age, sex, TIV, and BDI-II and BAI scores (*P* < 0.01, uncorrected) for each group; 43 significant differences were found between the SSD and normal control groups, and an association study was conducted for regions showing significance. As shown in Figure 4B, the SCL-90-R-SOM scores were negatively correlated with the rGMVs of right cerebellar lobe 3 and bilateral cerebellar lobes 4–5 in patients with SSD. Regarding the cognitive tests, TMT-A response time was negatively correlated with the rGMV of the right pallidum, while the CPT overt response score was negatively correlated with the rGMV of the left medial orbitofrontal gyrus (OFGmed), and the CPT response time was negatively correlated with the rGMV of the bilateral supramarginal gyrus (SMG) in patients with SSD.

### Comparison of Structural Covariance Between Somatic Symptom Disorder Subgroups

**FIGURE 5:**
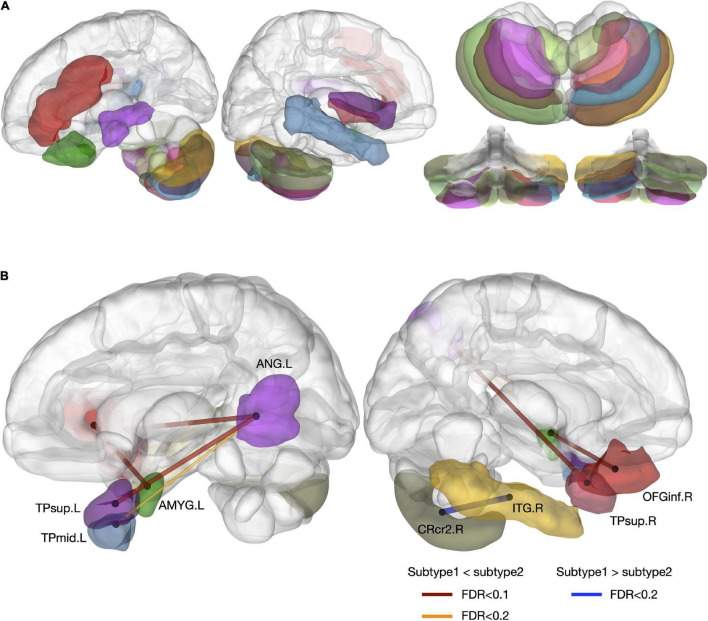
Differences in regional volumes and structural covariance networks between SSD subtypes. **(A)** Regional volume difference was reduced in subtype 2, compared to subtype 1 (FDR < 0.2). **(B)** Different structural covariance patterns between the two subtypes (FDR < 0.2). TPsup, superior temporal pole; AMYG, amygdala; ANG, angular gyrus; CRcr2, cerebellar crus 2; ITG, inferior temporal gyrus; OFGinf, inferior orbitofrontal gyrus; L, left; R, right.

Patients with SSD subtype 2 showed decreased rGMV at the left MFG, left inferior orbitofrontal gyrus (OFGinf), bilateral Rolandic gyri, right Heschl’s gyrus, right middle temporal gyrus (MTG), bilateral cerebellar crus 1, 2, 7b, 8, and 9, and left cerebellar region 10, compared to patients with SSD subtype 1 (FDR < 0.2; Figure 5A).

Greater structural covariance was detected between the bilateral TPsup and left angular gyrus, the left middle temporal pole (TPmid) and left angular gyrus, and the left amygdala and right OFGinf in patients with SSD subtype 2. In contrast, structural connectivity was greater between the right inferior temporal gyrus (ITG) and right cerebellar crus 2 in patients with SSD subtype 1 (FDR < 0.1–0.2; Figure 5B).

### Longitudinal Follow-Up

After 6 months of treatment, 23 patients (53.5%) underwent follow-up assessments; 3 received only non-pharmacological treatment and 20 received both pharmacological and non-pharmacological treatments (Supplementary Table 2). Table 2 shows the initial and follow-up clinical variables and cognitive functioning. The mean SCL-90-R-SOM (*p* = 0.001), PHQ-15 (*p* < 0.001), and BAI (*p* = 0.001) scores had significantly decreased at the 6-month follow-up assessment. There were significant changes in performance on the AVLT (*p* = 0.003) and VFT (*p* = 0.014). Spearman correlation analyses showed a significant association between Δ PHQ-15 and Δ VLT (*r* = −0.465, *p* = 0.025).

**TABLE 2:** The change of psychological and neurocognitive variables after 6 months in patients with SSD (*N* = 23).

	Initial	After 6-months		
	Mean (SD)	Mean (SD)	*t*	*P*
**Clinical variables**				
SCL-90-R-SOM	13.70 (8.11)	8.30 (4.56)	*t* = 3.87	0.001**
PHQ-15	13.13 (4.18)	8.83 (4.58)	*t* = 6.73	< 0.001***
BDI-II	16.30 (8.15)	13.13 (8.78)	*t* = 1.88	0.073
BAI	21.43 (12.75)	14.83 (11.11)	*t* = 3.69	0.001**
STAXI-state	12.43 (3.79)	12.13 (4.08)	*t* = 0.29	0.775
STAXI-trait	19.48 (5.19)	17.78 (4.50)	*t* = 1.72	0.100
TAS-20	54.00 (8.25)	50.00 (10.69)	*t* = 1.61	0.121
**Neurocognitive tests**				
Auditory Verbal Learning Test	55.00 (8.03)	59.74 (8.68)	*t* = -3.36	0.003**
Trail Making Test -A response time (s)	24.22 (7.60)	22.17 (7.01)	*t* = 1.53	0.141
Trail Making Test-B response time (s)	44.09 (16.72)	41.04 (18.64)	*t* = 0.99	0.333
Wisconsin Card Sorting Test	4.87 (1.82)	5.35 (1.61)	*t* = -1.25	0.223
Digit Span Test- forward	7.81 (0.81)	7.97 (0.62)	*t* = -1.63	0.118
Digit Span Test- backward	6.05 (1.50)	6.49 (1.20)	*t* = -1.87	0.075
Visual CPT- correct	134.26 (2.30)	134.35 (1.90)	*t* = -0.18	0.861
Visual CPT- overt	1.35 (2.71)	1.83 (2.53)	*t* = -0.89	0.382
Visual CPT- response time (s)	0.39 (0.06)	0.40 (0.05)	*t* = -0.71	0.485
Verbal Fluency Test	38.04 (11.32)	41.83 (13.19)	*t* = -2.66	0.014*
Verbal Fluency Test- repeat	1.13 (1.58)	1.04 (1.22)	*t* = 0.30	0.765

SSD, somatic symptom disorder; SD, standard deviation; SCL-90-R-SOM, Somatization subscale from Symptom Checklist-90-Revised; PHQ-15, Patient Health Questionnaire-15; BDI-II, Beck Depression Inventory-II; BAI, Beck Anxiety Inventory; STAXI-state, State-Trait Anger Expression Inventory-state; STAXI-trait, State-Trait Anger Expression Inventory-trait; TAS-20, Toronto Alexithymia Scale-20; TIV, total intra-cranial volume; CPT, Continuous Performance Test. All p-values are calculated with paired t-test (degree of freedom = 22 for all). *p < 0.05, **p < 0.01, ***p < 0.001.

However, regarding the rGMV and structural covariance, there was no significant changes in pre- and post-treatment comparisons. Also, correlations analyses revealed no significant association between Δ clinical variable and Δ rGMV.

## Discussion

### Reduced Structural Covariances in Patients With Somatic Symptom Disorder

In this study, we found altered structural covariances between cortical and subcortical regions in patients with SSD. Notably, the cerebellar-pallidal circuit showed the largest reduction in structural covariance in patients with SSD. Although the cerebellum has not been considered a central region in SSD, some studies have suggested cerebellar involvement in pain and somatization disorders (16, 19, 36). Other fMRI studies reported that activity in the cerebellar lobules was related to pain processing (37, 38), suggesting that the cerebellar network may be involved in the processing of chronic affective pain. According to a literature review, the pallidum was a key node in subcortical networks underlying somatosensory amplification, which plays a role in somatic symptoms (2). Guo et al. (39) were the first to report decreased functional connectivity of the pallidum in patients with somatization disorder. In line with previous findings, our study suggested that the set of reduced structural links underlying morphological alteration occurred solely at the cerebellar-pallidal circuit which has been associated with clinically relevant dysfunctions in SSD.

Functional imaging studies of SSD have also revealed involvement of various damaged networks, such as the DMN, SMN, salience network, and DAN (18). Consistent with these findings, regions showing reduced structural covariance were part of the DMN (TPsup and SFGdor) and SMN (Heschl’s gyrus and STG) in our study. Previous studies have suggested a role for the DMN in the loss of top-down regulation in neural mechanisms of SSD (17, 18). And interactions among the SMN, DMN and salience network appear to be associated with sensory processing, adjusted by attentional and emotional control in SSD (18, 40). Previous research reported abnormal functional connectivity in the bilateral MOG of patients with SSD and suggested that activity in this region may be associated with aversive experiences and somatic symptoms (36) and the dorsolateral prefrontal cortex (DLPFC) including SFGdor is thought to subserve top-down attentional control (41) and the inhibitory control of pain processing (42). Although the present results could not explain how these reduced covariances between regions are related to the mechanisms of SSD, results suggest the existence of covarying structural links that were previously demonstrated as core functional networks in SSD.

### Clinical Symptoms and Neurocognitive Functioning in Somatic Symptom Disorder

In the present study, regional volumes of several regions were related to clinical and cognitive dysfunctions in patients with SSD. SCL-90-R-SOM scores were negatively correlated with the rGMVs of the right cerebellar lobe 3 and left cerebellar lobes 4–5; thus, a smaller GMV of the regional cerebellum was associated with more severe somatic symptoms. This result of the involvement of the cerebellum and its association with somatic symptoms in SSD as shown in fibromyalgia (43) support the presence of common neural involvement as well as similar clinical symptoms in these diagnoses. On cognitive tests, patients with SSD showed significantly poorer performance than did the normal controls on the tests of verbal functioning and sustained attention. Positive associations of the left OFGmed and bilateral SMG GMV with sustained attentional capacity, as measured by the CPT, were observed in this study. The OFGmed and SMG were shown to be involved in selective attention (44), and previous studies demonstrated impaired attention and attentional bias in SSD patients, who tend to be hypervigilant to somatic sensations, rendering them less responsive to external stimuli (45). Impairment in these regions could reduce the capacity for sustained attention, as observed in patients with SSD. Correlation analyses indicated that patients with a smaller rGMV of the right pallidum performed worse on the TMT-A, which measures attention and psychomotor speed. Performance on the TMT-A mostly reflects motor skills, which involve basal ganglia–thalamocortical circuits (46); this may explain the link between TMT-A performance and pallidum volume. In summary, these results suggest that the right pallidum, left OFGmed and SMG may be relevant structural regions related to neurocognitive functioning in patients with SSD.

### Specific Structural Regions in Somatic Symptom Disorder Subtypes

In our study, patients with pain exhibited lower GMV in the left MFG, left OFGinf, bilateral Rolandic gyrus, right Heschl’s gyrus, right MTG, and cerebellum, compared to patients with dizziness. Moreover, significant structural connectivity was observed between the bilateral TPsup and left angular gyrus, left TPmid and left angular gyrus, and left amygdala and right OFGinf in patients with pain. These regions are mainly involved in the DMN (i.e., the MFG, OFG, MTG, and angular gyrus), salience network (i.e., the amygdala), and SMN (i.e., the Heschl’s gyrus, Rolandic gyrus, and cerebellum), which are key damaged functional networks consistently observed in SSD and pain disorders (16, 18). These regions are known to be involved in memory and emotional tagging of sensory perception, suggesting that emotional responses to pain inputs may alter the intensity and experience of pain symptoms (47). Meanwhile, patients with dizziness exhibited greater structural connectivity between the right ITG and right cerebellum in the dominant hemisphere (with respect to vestibular functioning) in right-handed individuals. The ITG is involved in visual perception, language, and memory previous studies reported the relationship of the ITG to alexithymia and somatization disorder (39, 48). Increased structural connectivity between the ITG and cerebellum would enhance the perception and memory of dizziness, which might underlie the maladaptive cortical plasticity and memory consolidation seen in patients. We included patients with dizziness in this study, which is a relatively understudied symptom compared to pain in SSD. Furthermore, dizziness has been placed at the interface of functional neurological disorder and SSD; these diagnoses are defined using similar characteristics, and share some pathophysiology (49). Our findings suggest that specific structural alterations are involved in specific symptom presentations in SSD that differ from the neural circuits common to core psychological symptoms of SSD. The present results elaborate further studies related to subtyping of SSD to understand diagnostic clarification and interventions more fully.

### Longitudinal Changes After Treatment

After 6 months of treatment, patients with SSD showed a significantly improved performance on verbal memorization and verbal fluency as well as reduced somatic symptoms compared with initial assessment. The changes of cognitive functioning after treatment may reflect improved clinical status. Specifically, the score on VFT of the patients was significantly lower at initial assessment compared with the normal controls but recovered after treatment. The correlation between the changes of PHQ-15 score and VFT score further support that specific cognitive functioning is associated with the disease state. Although the data on neurocognitive dysfunction in SSD are inconsistent, the neurocognitive functions of attention and verbal memory have repeatedly been suggested to be possible neurobiological markers that can be impaired in patients with SSD (7, 8). In a previous work, we also introduced that neurocognitive dysfunctions are subtle, but certain cognitive functions may be related to the somatic symptoms and clinical improvements of patients with SSD (50).

In this study, there was no significant differences in the GMVs between patients with SSD and normal controls, though the GMVs of several regions were associated with clinical symptom severity. Our results may suggest candidate regions relevant to somatic symptom and cognitive functioning rather than specific structural alterations involved in the development of SSD. Results of the previous structural studies in SSD were inconsistent, which have reported morphological alterations in the caudate, ACC, MFG, angular gyrus, pituitary, amygdala, hypothalamus, fusiform gyrus, cuneus, inferior frontal gyrus, PCC and cerebellum (12–14). However, a recent review pointed that previous studies of SSD were rarely account for the influence of psychiatric comorbidities, thus observed structural changes may be related to comorbid psychiatric disorders rather than to SSD itself (51). Indeed, a previous study found no volumetric differences between patients with SSD and normal controls when controlling for depression and anxiety (52, 53). These discrepancies may also be present due to heterogenous population with various symptoms, differences in disease duration and the impact of inconsistent treatments. Additionally, we found no significant changes of the GMV and structural covariance in patients with SSD after treatment regardless of clinical improvement. These findings may support that SSD is related to altered brain networks rather than altered regional structures. Previous studies have suggested that pain causes network dysfunctions rather than structural abnormalities (54, 55). A recent study revealed altered structural covariance network but no differences in structural volumes in patients with headache compared with normal controls (56). Another longitudinal study reported that there was no longitudinal GMV alterations in patients with migraine, which suggests that migraine is not a structural disease and the use of network analyses on functional imaging may show rather robust findings in migraine (57). Therefore, further studies using combined neuroimaging modalities may clarify how structural and functional networks are related and engaged in SSD.

Taken together, we can assume that SSD is a disease with dysfunctions of complex brain networks rather than defects of regional structures, and the structural covariance rather than regional volume may capture these alterations and altered structural covariance may interact with a risk or vulnerability trait rather than a disease state. Longer period of observational study with a larger sample size is needed to examine relationships among these factors.

This study has several limitations. First, we could not completely rule out effects of depression and anxiety on clinical symptoms or cognitive functioning, although the BDI-II and BAI scores were used as covariates in the analyses. Also, we did not include factors related to social functioning, which are associated with clinical symptoms. Second, due to the modest sample size we could not perform correlation analyses between clinical variables and imaging data within subgroups. In our study, correlations between structural covariances and clinical variables were not significant after FDR correction, and we presented them with uncorrected *p*-values. This may also be due to the restricted sample size and we recommend future studies with larger sample sizes. Third, all images were preprocessed and analyzed by independent researchers who were blinded to the patients’ clinical data. However, investigators were not blinded to group allocation for performing neurocognitive tests and statistical comparisons. These may have led to assessor bias. Lastly, though this longitudinal study was preliminary a drop-out rate was relatively high, and the follow-up assessments were performed on only SSD patients; thus, it was not possible to examine a group × time interaction.

## Conclusion

To the best of our knowledge, this study is the first to explore structural alterations and their relationships with organ symptoms and neurocognitive functioning of SSD using structural covariance analysis. Also, this is the first longitudinal structural MRI study of SSD. Ethno-genetic studies have suggested that Asians more frequently exhibit somatic symptoms compared to members of Western cultures, and patients in Asian countries more often complain of breathlessness and dizziness (58). The present investigation of brain structural patterns in SSD in this population may also provide additional understanding of the cross-cultural specificity of this disorder. This study demonstrated structural alterations in the pallidal-cerebellar circuit, as well as in the DMN and SMN, in patients with SSD. Correlations were observed between rGMVs and clinical/neurocognitive variables in patients with SSD, which suggests the involvement of structural alterations in SSD. Meanwhile, structural alterations in GMV were subtle in patients with SSD despite their experiencing definite discomfort from somatic symptoms. This might suggest the presence of neural alterations at the level of “functional” rather than “structural” disruptions in SSD that are relevant to “dysfunctional” rather than “impaired” states. Our results regarding alterations in structural covariance other than in rGMVs may support this and suggest the utility of structural covariance analysis combined with functional imaging to explore attenuated structural alterations in this population. Notably, our longitudinal observation of structural covariance in clinically improved patients suggest that attenuated structural covariance may reflect the risk or vulnerability to SSD rather than clinical courses. Additionally, subtype analyses suggested that specific neural networks and brain regions may contribute to the presentation of organ phenotypic symptoms among various somatic symptoms in SSD but this hypothesis should be tested with detailed experiments in further studies. Also, further studies including other SSD subtypes, such as patients with gastrointestinal and cardiopulmonary symptoms, may facilitate the elucidation of brain networks underpinning the subtypes.

## Data Availability Statement

The raw data supporting the conclusions of this article will be made available by the authors, without undue reservation.

## Ethics Statement

The studies involving human participants were reviewed and approved by the Institutional Review Board of Seoul National University Bundang Hospital (B1710426302). The patients/participants provided their written informed consent to participate in this study.

## Author Contributions

HP: funding acquisition, conceptualization, data curation, and writing – preparation of the original draft. YJ: data curation and analysis of data. LS: data curation and analysis of imaging data. I-YY: conceptualization and methodology. BP: methodology, statistical analysis, and figures. All authors contributed to the article and approved the submitted version.

## Conflict of Interest

The authors declare that the research was conducted in the absence of any commercial or financial relationships that could be construed as a potential conflict of interest.

## Publisher’s Note

All claims expressed in this article are solely those of the authors and do not necessarily represent those of their affiliated organizations, or those of the publisher, the editors and the reviewers. Any product that may be evaluated in this article, or claim that may be made by its manufacturer, is not guaranteed or endorsed by the publisher.
