# Supplementary material for: A Longitudinal Study on Attenuated Structural Covariance in Patients With Somatic Symptom Disorder
Source: Front Psychiatry. 2022 May 17;13:817527. doi: 10.3389/fpsyt.2022.817527 (PMC9152139; doi:10.3389/fpsyt.2022.817527)
Supplement: Supplementary file 1 [file Data_Sheet_1.doc]

**Table S1. Structural covariance connectivity changed in somatic symptom disorder group**

| Connections | | SSD (N = 43) | | | Normal Control (N = 30) | | | Comparisons | |
| --- | --- | --- | --- | --- | --- | --- | --- | --- | --- |
| Region | Region | Structural covariance (r) | | *p* | Structural covariance (r) | | *p* | *p*  (uncorrected) | FDR-corrected *p* |
| CRcr2.L | PAL.L | -0.323 | 0.0476 | | 0.562 | 0.0035 | | 1.05×10^-4^ | 0.071 |
| CRcr2.R | PAL.L | -0.369 | 0.0226 | | 0.535 | 0.0058 | | 8.56×10^-5^ | 0.064 |
| CR7b.L | PAL.L | -0.424 | 0.008 | | 0.551 | 0.0043 | | 2.16×10^-5^ | 0.029 |
| CR7b.R | PAL.L | -0.414 | 0.009 | | 0.623 | 8.8×10^-4^ | | 3.95×10^-6^ | 0.018 |
| CR8.R | PAL.L | -0.312 | 0.0563 | | 0.525 | 0.0071 | | 2.74×10^-4^ | 0.150 |
| CRcr2.L | PAL.R | -0.276 | 0.0932 | | 0.638 | 0.0006 | | 3.74×10^-5^ | 0.041 |
| CRcr2.R | PAL.R | -0.320 | 0.0505 | | 0.632 | 0.0007 | | 2.05×10^-5^ | 0.029 |
| CR7b.L | PAL.R | -0.410 | 0.0106 | | 0.572 | 0.0028 | | 1.73×10^-5^ | 0.029 |
| CR7b.R | PAL.R | -0.359 | 0.0269 | | 0.650 | 0.0004 | | 5.63×10^-6^ | 0.018 |
| CR8.L | PAL.R | -0.365 | 0.0243 | | 0.500 | 0.0108 | | 1.87×10^-5^ | 0.114 |
| CR8.R | PAL.R | -0.315 | 0.0541 | | 0.583 | 0.0022 | | 7.65×10^-5^ | 0.064 |
| CR8.R | CRcr1.L | 0.265 | 0.1072 | | 0.861 | 3.4×10^-4^ | | 4.63×10^-5^ | 0.044 |
| TPsup.R | SFGdor.L | -0.118 | 0.4807 | | 0.652 | 0.0004 | | 3.11×10^-4^ | 0.150 |
| PRCU.R | MOG.R | -0.005 | 0.9754 | | 0.699 | 0.0001 | | 4.45×10^-4^ | 0.199 |
| STG.L | HES.L | 0.414 | 0.009 | | 0.871 | 1.5×10^-4^ | | 3.13×10^-4^ | 0.150 |

SSD, somatic symptom disorder; PAL, pallidum; CRcr2, cerebellar crus 2; CR7b, cerebellar lobe 7b; CR8, cerebellar lobe 8; CRcr1, cerebellar crus 1; TPsup, superior temporal pole; SFGdor, dorsal superior frontal gyrus; PRCU, precuneus; MOG, middle occipital gyrus; STG, superior temporal gyrus; HES, Heschl’s gyrus; L, left; R, right.

**Table S2. Treatments in patients with somatic symptom disorder who were followed for 6 months**

| Number | Age | Sex | Subtype | Treatment |
| --- | --- | --- | --- | --- |
| 1 | 38 | F | 1 | Escitalopram, Clonazepam, Supportive psychotherapy |
| 2 | 58 | M | 2 | Amitriptyline, Relaxation therapy |
| 3 | 63 | F | 2 | Amitriptyline, Clonazepam, Relaxation therapy |
| 4 | 42 | F | 2 | Relaxation therapy, Supportive psychotherapy |
| 5 | 37 | M | 2 | Duloxetine, Alprazolam, Relaxation therapy |
| 6 | 53 | M | 1 | Escitalopram, Clonazepam, Supportive psychotherapy |
| 7 | 40 | F | 2 | Amitriptyline, Supportive psychotherapy |
| 8 | 63 | F | 2 | Duloxetine, Relaxation therapy |
| 9 | 64 | F | 2 | Milnacipran, Amitriptyline, Supportive psychotherapy |
| 10 | 49 | F | 1 | Escitalopram, Alprazolam, Supportive psychotherapy |
| 11 | 46 | M | 2 | Escitalopram, Trazodone, Relaxation therapy |
| 12 | 46 | F | 1 | Escitalopram, Clonazepam, Supportive psychotherapy |
| 13 | 45 | F | 1 | Sertraline, Alprazolam, Supportive psychotherapy |
| 14 | 51 | F | 2 | Amitriptyline, Clonazepam, Relaxation therapy |
| 15 | 31 | M | 1 | Melatonin, Supportive psychotherapy |
| 16 | 48 | F | 1 | Escitalopram, Diazepam, Supportive psychotherapy |
| 17 | 60 | F | 2 | Amitriptyline, Relaxation therapy |
| 18 | 39 | F | 2 | Relaxation therapy, Supportive psychotherapy |
| 19 | 41 | M | 1 | Escitalopram, Clonazepam, Supportive psychotherapy |
| 20 | 30 | F | 2 | Escitalopram, Amitriptyline, Alprazolam, Relaxation therapy |
| 21 | 58 | M | 2 | Amitriptyline, Relaxation therapy |
| 22 | 36 | M | 2 | Escitalopram, Duloxetine, Relaxation therapy |
| 23 | 24 | F | 2 | Relaxation therapy, Supportive psychotherapy |
